# Preparation and use of plant medicines for farmers' health in Southwest Nigeria: socio-cultural, magico-religious and economic aspects

**DOI:** 10.1186/1746-4269-6-1

**Published:** 2010-01-20

**Authors:** Taiwo E Mafimisebi, Adegboyega E Oguntade

**Affiliations:** 1 Department of Agricultural Economics and Extension, School of Agriculture and Agricultural Technology, The Federal University of Technology, Akure, Nigeria

## Abstract

Agrarian rural dwellers in Nigeria produce about 95% of locally grown food commodities. The low accessibility to and affordability of orthodox medicine by rural dwellers and their need to keep healthy to be economically productive, have led to their dependence on traditional medicine. This paper posits an increasing acceptance of traditional medicine country-wide and advanced reasons for this trend. The fact that traditional medicine practitioners' concept of disease is on a wider plane vis-à-vis orthodox medicine practitioners' has culminated in some socio-cultural and magico-religious practices observed in preparation and use of plant medicines for farmers' health management. Possible scientific reasons were advanced for some of these practices to show the nexus between traditional medicine and orthodox medicine. The paper concludes that the psychological aspect of traditional medicine are reflected in its socio-cultural and magico-religious practices and suggests that government should fund research into traditional medicine to identify components of it that can be integrated into the national health system.

## Introduction

Health, wellness and economic growth are important in the overall development of a people. A state of health is said to exist when there is perfect harmony between humans and their environment. As a step forward to the desired goal of health for all in 2015, there is increasing interest in the development and use of products of traditional medicine (TM) [1]. Africans even in urban areas often 'supplement' the care they receive in clinics and hospitals with treatment from traditional healers [2,3]. It is estimated that traditional medicine provides 80%-90% of healthcare in Africa [1].

Nigeria, like any other typical African country, is an agrarian economy in which agriculture and agro-allied enterprises are the most popular income-generating activities providing employment for up to 90% of the rural dwellers [4]. The rural populace, which constitutes about 70% of the country's total population and provides virtually all of the nation's home-produced food, usually has little or no access to quality orthodox medicine (OM). In addition, disease incidences are higher in the rural areas because of higher levels of illiteracy, poverty and ignorance [5]. These rural dwellers rely almost exclusively on TM for their healthcare needs in order to remain economically active. Were it not for TM therefore, it is probable that the food problem in Nigeria would have been more acute than it presently is [4,6-9].

According to WHO [1], TM is the sum total of all knowledge and practices, whether explicable or not, used in diagnosis, prevention and elimination of physical, psychological, mental or social diseases relying exclusively on past experience and observations handed down from generation to generation whether verbally, in writing or by other means. Awah [3] extended this definition by stating that TM "refers to practices and approaches that apply - separately or in combination - plant-, animal- and mineral-based medicines, spiritual therapies, manual techniques and exercises to diagnose, prevent and treat illnesses, or maintain or enhance well-being". For Africa, TM is a holistic concept which covers nature, the sociological environment whether living or dead and the metaphysical forces of the universe [10-12]. A further extension defines TM "as alternative or non-conventional modes of treatment often involving the use of herbs in a non-orthodox manner as well as the process of consulting herbalists, mediums, priests, witchdoctors, medicine men and various local deities when seeking a solution to diverse illnesses "[13]. TM, often referred to as indigenous medicine in Asia, is as old as creation itself. The importance of plants for medicinal purposes is revealed in many verses of the Bible among which Ezekiel 47:12 ''the fruit of trees shall be for eating and the leaves for medicine" is very notable. Both plants and animals have been used as sources of medicine since pre-civilization period [14-16]. Even in modern times, animals and plants continue to play an indispensable role in healthcare [17,18]. Plant and animal parts form important ingredients in preparing medicines which can be curative, protective or preventive [19]. Thus, a considerable percentage of currently available non-synthetic and/or semi-synthetic pharmaceuticals used in OM comprises of drugs originating from higher plants [20,21] followed by microbial, animal and mineral products [22]. OM is defined as medicine based on scientific methods and taught in western medical schools [13]

From time immemorial, Nigerians depended almost exclusively on TM for their healthcare needs and there were enough plants to sustain its practice. There was no record of OM practice in Nigeria before 1873 when the Lagos General Hospital was established. Following this, a make-shift temporary civil hospital was built in Asaba (now in Delta State of Nigeria) in 1888. A Government Hospital was also built in Calabar in 1898 as a result of the wide impact the first two hospitals made on the indigenes and the colonial personnel and their families. As OM received official and government promotion and funding, people were made to believe that it was a better alternative to TM. TM was thereafter adjudged by the colonial authorities to be dangerous and inimical to health. Sermons by some sects in the Christian religion also equated TM to idol worship [2,23]. Furthermore, some deficiencies inherent in the practice of TM such as lack of standardization of the prescribed dosage are making a growing number of elites to disdain it. As a result, the practice of TM was completely left unorganized and scientifically undeveloped.

## Socio-cultural and magico-religious practices in plant medicines preparation and use

TM like OM aims at healing or preventing diseases. In this respect, there is a congruence of objective between both types of medical systems with the point of departure being their concept of the causes of diseases, approach to healing and healing method used. The basic concept of OM centres on the result of experiments, and diseases that are regarded as being caused by physio-pathological agents. TM however considers humans as an integral somatic and extra-material entity and in many developing countries, it is accepted that diseases can be due to supernatural causes arising from the displeasure of ancestral gods, evil spirits, evil wind, the effects of witchcraft, the effects of spirit possession or the intrusion of an object into the body. TM is therefore a system which places greater emphasis on psychological causes of disease than does OM [3,10,13,24].

Furthermore, TM is part of the culture of the people that use it and as a result, it is closely linked to their beliefs. Majority of the practitioners of TM in Nigeria are people without western education who had the practice handed down to them by their forefathers through informal training, verbal communication and folklores. Others acquire their capabilities through dreams and sometimes, supernatural forces [10-12,24]. Therefore, embedded in TM are some socio-cultural and supernatural or magico-religious practices. It is believed that the magico-religious practices must be observed for medicinal preparations to be potent. It is also widely held by the practitioners that the non-observance of these magico-religious practices will cause the medicines not to produce the desired effects. In addition, the practitioner may incur the wrath of the gods and may, as a consequence, suffer some calamities either mild or severe.

Some of these practices are considered in some detail below. Where possible, scientific explanations are advanced for some of them and comparisons are made with what is obtainable in OM to clearly bring out the nexus, if any.

1. TM practitioners often observe the physical and biochemical reactions of plants and interpret them as esoteric phenomena [10,25]. An example is a plant called touch -me -not, *Mimosa pudica; *which closes up when touched [26] and pitcher-an insectivorous plant-which has a mechanism for trapping and enzymes for digesting insects which land on it for nectar collection. TM practitioners think that plants sleep during the night and are awake during the day. This is their interpretation of the physiological and bio-chemical processes going on within plants.

2. Arising from the perception that plants sleep in the night and are awake in the day, TM practitioners believe that for some medicines to be potent, collection of whole plant or plant parts for medicinal preparationsshould be made before 6.00 pm in the evening. The reason given for this by practitioners is that 'the forces which produce active ingredients that make plant medicine potent and efficacious would have gone to rest by evening time. Considering the fact that these practitioners do not know about photosynthesis and the active ingredients which it yields in plants, one will be forced to respect their opinions and beliefs about plants [12]. Elementary science confirms that the leaves of a potted plant that is removed from sunlight for up to 24 hours will test negative for the presence of starch [27]. The practice of fetching plant leaves for some medicinal preparations before sunset is a cultural belief by practitioners of TM that scientific evidence supports.

Sometimes, the practitioners specify that a particular plant part to be used for some medicines be collected at specific time of the night for it to have the desired efficacy. This practice cannot be dismissed as totally absurd when it is considered that there is diurnal variation in the concentration of some plant constituents [10,28]. The practitioners may have found that collections made at night are more effective than those made during the day for some plants through repeated preparation of the same medicine with plant parts collected at different times. For example, the volatile oil-containing Siam weed (*Chromolaena odoratum *L.) loses its oil content probably due to evaporation, in bright sunlight, but the concentration is at a peak from sunset to midnight [29]. It may also be for increased concentration of active components that some flowers are more flagrant at night compared with the day [10].

In the preparation of certain traditional remedies, TM practitioners specify that the dead leaves that have fallen off the plant be picked for use rather than the green leaves still turgid on the tree. This is usually the case with paw-paw (*Carica papaya *L.), banana (*Musa spp*.), orange (*Citrus spp*.) and Mango (*Magifera indica*) leaves. According to the practitioners, the dead leaves are usually brown and richer in some active agents than the green leaves. They also assert that the plant would have passed into the dying leaves, certain unwanted metabolites which are required for the medicine. Scientists have confirmed that the shed leaves of some plants are richer in phenolic substances and waste metabolites [30].

In some other cases, fresh young leaves rather than the older leaves are specified for use in TM. For a mild purgative, for example, it is the young leaves of *Cassia alata *L. that are prescribed. Young leaves of plants are known to be richer in certain constituents than the old leaves [30]. Some transformations of the constituents take place in the leaf as it grows older. For example, the young tiny leaves of *Ocimum gratissimun *are richer in essential oil than the older, bigger leaves [10]. Thus, it is obvious that the uneducated TM practitioners know where to get the active principles needed for their medicinal preparations in their highest concentrations without the knowledge of western science. Another interesting differentiation of medicinal plants is in terms of location. For example, TM practitioners in Nigeria recommend the mistletoe that is growing on cocoa trees to be used for medicinal preparation for the treatment of hypertension as opposed to the same plant growing on oil palm or any other tree.

3. In some cases, during the process of harvesting whole or plant parts for TM, practitioners sing the praises of the *gods *that gave these plants for human use and the *'strong and awakening spirits' *working in these plants to produce efficacy when they are used as sources of medicine. This is done by chanting incantations which are a set of practical real life facts arranged in poetic and rhythmical manner such that it becomes pleasant to the ear. Sometimes, an incantation takes the form of a play on words (pun) delivered orally in poetic form apparently to induce the spirit controlling a particular plant to conjure up efficacies into the part to be used for preparation of medicine [10]. This practice is akin to the recitation of certain portions of the Bible or Quoran or other religious books in faith healing in many parts of the world [31].

Incantations are not limited to the time of procuring materials to be used for medicine alone. They are said at different stages (during the arrival of a patient, diagnosis of ailment, preparation of medicament, administration of medicament, after signs of healing have been noticed and during the discharge of a patient). Some incantations said when preparing a medicine are all encompassing including naming all the components of the medicinal preparation and the roles that they are supposed to play [10]. By the play on words, parts of the names of the constituents are transformed in the verse to active effects desired of the medicine as shown in the following example by Verger [32]:

   *Ewe ooyo aje, ba wa yo arun kuro ni iha*

      *Ewe awusa, sa arun iha kuro*

         *Iye agbe, gbe arun iha kuro*

         *Iye aluko, ko arun iha lo*

Translation:

            *Ooyo aje leaf chase away (yo) for us the disease out of the flank*

            *Awusa leaf heal (sa) the flank disease so it may go away*

            *Agbe feather carry (gbe) the flank disease off*

            *Aluko feather pick (ko) away the disease out of the flank*

Another popular example is

            *Ohun ti a ba wi fun ogbo ni ogbo n gbo*

               *Ohun ti a ba wi fun ogba ni ogba n gba*

                  *Ohun ti mo ba wi fun iwo lagbaja ni kio gba*

Translation:

Whatever we tell ogbo (the leaf of Parquetina nigrescens Baill) it hears (gbo) or does not refuse

Whatever we tell ogba (a fenced precinct of a domicile) it accepts (gba)

*Whatever I tell you (any individual named here) you must accept (gba)*.

As observed by Sofowora [10], it is erroneous to assume that incantatory effects depend exclusively on the alliterative nature of the verses, for there are verse forms (at least in the Yoruba language) that are heavily alliterative but not incantatory. The efficacy of an incantation is derived from it evocative power. Thus, if a practitioner of TM invokes the 'original' name i.e. from 'the source or the circumstances of the existence of a particular being (deified or not) into a medicine, the medicament takes on magical or conjuring forces.' For example, a violent lunatic may have to be induced into deep sleep through incantations so that treatment can be administered. Also, when an attacker or assailant attacks a practitioner of TM unawares especially in a secluded place, the first and most practicable defense is the use of incantations which can hypnotize the attacker and lead to his/her capture or withdrawal [33].

It is believed that some traditional medicines can only be effective when an incantation is recited during their preparation and administration. The incantation and the potion together form the treatment and neither alone can effect a cure or elicit the desired response. It should be noted that the effect or function of an incantation in producing a cure in TM cannot be easily proved experimentally [11]. For this reason, ethno-medical research in many institutions has been limited to those aspects of traditional healing methods which do not involve incantations. The more powerful the medicine or the situation to be controlled is, the more the need for incantations. Incantations are practiced to an extent that one can easily believe the claim by some TM practitioners that a supernatural power exists. Some scientists have been tempted to group incantations into the esoteric area of the occult or trans-physical science. They see it neither as a placebo effect nor mere magic [10]. Incantation chanting is not limited to Africa as it is also said to be practiced by the aborigines of Australia, Amerindians and various Asiatic peoples [10,34].

4. Consultations before admitting patients or commencing treatment are common in TM. Sequel to the belief that diseases can be caused by supernatural forces [3], a TM practitioner may consult an oracle or the gods about the patient to find out the cause of the disease and the appropriate remedies and treatment. If the practitioner receives a favourable response, s/he admits the patient and commences treatment otherwise the patient and the people that brought him/her are turned down. The purpose of the consultation is to seek the express permission from the gods to treat especially sub-somatic cases.

The art of diagnosis through oracle is common in Nigeria using the 'Ifa oracle' priests. The TM practitioners (over 80% of which has knowledge of consultations using the Ifa oracle) throw the "opele" (a stringed object of divination) on a wooden tray and each arrangement of the "opele" is characteristic of a specific "Odu" or chapter in a series of poems or verses which the priest or TM practitioner has learnt during his/her training. By reciting the appropriate "Odu" corresponding to a particular pattern, it is believed that the story told in the chapter (of which there are 256 indicates the illness of the patient as well as the remedies and sacrifice(s) to be made [35]. Variations in this theme are known all over Africa [10].

Another form of diagnosis is done through trance. TM practitioners possessing this ability enter into a trance as soon as a patient arrives or when people come to consult them about an ancestral problem. While s/he is in a trance, the words spoken by the practitioner are noted and usually the callers (patients and the people that brought them) give a positive sign or response to let the practitioner know whether or not s/he is giving a correct diagnosis of the problem. This ability is used by TM practitioners not only to identify an illness but also to prescribe an appropriate treatment for it. In some cases, the practitioner can actually communicate with spirits in her/his trance. In this case, s/he will attempt to link up with the spirit of the person who may be responsible for the problem or knows of its cause. Through the practitioner, the spirit narrates what is wrong, as well as the sacrifices necessary to appease the gods [10].

Another form of diagnosis is watching from a mirror or a pot filled with water, which, after been ordered to do so through incantations, serves like a television screen by transmitting the medical history of the patient and the series of events leading to the present ailment. The *dramatis personae *of this spiritual "film or movie" are carefully noted for their roles. After this, the practitioner, in cases of sub-somatic diseases, links up with the spirit of the patient or that of his ancestors through praise-singing incantations and appeasing sacrifices to help suggest the treatment. The cure to some ailments may be appeasing living or dead persons who have been offended by the action or inaction of the patient [3].

5. Arising from point number 4 above, the gods or Ifa oracle may reveal to a TM practitioner certain conditions which must be observed during preparation of medicament and treatment of any complex case brought to him/her. S/He might for example be informed that s/he cannot fetch the components of the medicament by him/herself. S/He might be told to send a young boy or girl who is a virgin or who is "clean" (free of any manipulations by evil spirits). In this particular case, the practitioner may accompany the young boy or girl to the various places where the materials for the medicine may be found and actually show it to him/her, but s/he would have to allow the errand boy or girl to fetch the materials. Sometimes, the errand boy or girl will be required to keep mute until the materials have been delivered to the practitioner in his/her herbal home. If the "keep mute" order is violated, the fetched plant part is discarded as it is believed that medicine prepared from it cannot be efficacious. The process of fetching plant parts will then be repeated. The practitioner or the patient might also be told to abstain from sex, certain food items and avoid going to specified places for a specified time period till the patient had started showing signs of recovery. It is believed that the spirits controlling some plants and ailments are very sensitive to uncleanness and desecration and could prevent the efficacy of some components of TM if the conditions stated above are not strictly adhered to. The consequences are that the medicine will not be efficacious and the practitioner may also be punished in several ways for disobeying the instructions of the gods.

6. The number of various materials fetched to form components of a medicine is specific for male and female at least in the Yoruba speaking areas of Nigeria. While for males the number is nine or a multiple of it, for females, the number is usually seven or multiples of seven. The same number of nine and seven are applicable to male and female children, respectively. Thus, if the leaves of certain plant species are to be fetched for decoction, concoction or cold extract or incisions are to be made on certain parts of male or female bodies, for application of medicament, the practitioner is required to hold tenaciously to the number for the respective sex or the treatment is rendered inefficacious.

7. Ritual rites are also common with TM especially in the treatment of complex ailments such as barrenness, mental disorder, leprosy and consistent ill luck or calamity. Ritual rites are forms of procedure and/or sacrifice necessary to appease the gods in a particular form of treatment or situation. Rituals can involve presentation of cooked or raw foodstuff with money or local gin, sacrificing an animal or performing certain dances at specified time of the day or night at designated places usually a shrine, groove, forest, T- or X-junction or near a river. These locations are believed to be the habitation for ancestral spirits. Instructions for efficacy of rituals can also include eating only certain foods or parts of food and abstaining from certain things. Ritual rites are usually carried out by the patient or a specified relative of the patient under strict guidance by the TM practitioner to ensure compliance. In order to ward off the spirit of smallpox in a village, ritual rites are often thought to be necessary. Sacrifices are placed at various points around the village supposedly to keep the evil spirits from entering the village or to drive them away and prevent them from returning. These rites create the right milieu for the traditional treatment of which they are part, and no TM practitioner would risk waiving them. Ritual rites can be for protective, preventive or curative purposes [10,36].

8. Although, a traditional medicament may contain only a single active item, it is often a multi-component mixture that is prepared and administered and the practitioners have a reason for this. Some of the components function as preservatives, others do the work of flavouring agents while others are colourants. The practice of adding non-medicinal components to a preparation is not uncommon in modern pharmaceutical practice, where paediatric preparations, for example, are not only flavoured but are also coloured. Multi-component preparations are also preferentially prescribed in TM because in a single decoction, the patient is treated for all his ailments. This contrasts sharply with OM where the patient may have three or four different tablets/capsules to take, as well as receiving an injection. Both TM and OM methods of prescription practice can result in drug interaction problems. Whereas in the traditional decoction, drug-drug interaction takes place in the cooking pot, in OM practice, mixed drugs are more likely to interact inside the patient's stomach, if the doctor or prescriber does not take adequate care [10].

9. TM products can be prepared in several forms. They can be liquid (e.g. decoctions, infusions, oily mixtures, gargles etc), solids (e.g. powders for internal administration with hot maize pap or other drinks), semi-solids (e.g. certain crude balsams, resins, latex) or gaseous (e.g. steam inhalation preparations, fumigations like incense, etc). Intra-uterine mode of drug administration is also applied in the cure of certain STDs and in induced abortion. The intravenous route of administration is conspicuously absent in the application of TM. The equivalent of subcutaneous injections or vaccinations in OM is also found in TM. Incisions are made on the skin (face, chest, ankle, back or sides) with a razor blade or a sharp object and a powdered drug is rubbed into the incision, presumably to allow direct absorption of the active constituents of the drug through the capillaries. The incisions (1-2 cm long) are usually deep enough to cause bleeding [37]. The drug which is rubbed into the incision is usually made by burning various herbs together giving an almost charcoal-like product. This may be a way of concentrating the active ingredients which is absorbed onto the charcoal residue. The charring is however also capable of causing degradation or decomposition of any active principles in the plant material. External applications include traditional cosmetics (coconut oil, palm kernel oil) as well as dermatological preparations dissolved into these oils, which are rubbed onto the skin.

10. TM practitioners also practice specialization and referral health system. A practitioner will at first accept all non-complex cases and some complex cases after consultations. If however there are no noticeable signs of recovery by the patient after some trials in a complex or non-complex case, the practitioner will refer the patient to another practitioner who is a specialist in that particular ailment. If the family of the patient gives their consent, the patient is moved to the specialist immediately. Also, where the leaves for producing a particular medicament is not available in the community of the TM practitioner, s/he sends a message or goes personally to another practitioner in a near-by community to get the leaves or the medicament which is already made by another practitioner. Instances abound now in which TM practitioners refer cases to OM practitioners especially where s/he is aware that one exits nearby. OM practitioners, who have started to appreciate and acquire knowledge from TM practitioners, are also known to discharge their patients advising that the relatives seek help from TM practitioners. They do this once they are convinced that a medical case goes beyond the confines of OM. This is a good development to support the call for integration of the two medical systems in Nigeria.

11. Finally, in recognition of the fact that over-harvesting of plants for TM impacts negatively on the ecosystem and may lead to extinction of extremely important medicinal plants, certain cultural practices of TM practitioners in particular and indigenous people in general, promote conservation of plants [2]. The cultural taboos in some ethnic groups are compatible with the principle and practice of conservation of medicinal plants. Among the Yoruba and many other ethnic groups of Nigeria and Ghana, it is generally believed that the forest is not only a gift from the ancestors but also their abode and so must not be set on fire [38,39]. Any person who commits such a great offence even if inadvertently, is expected to own up and present specified animals to the priest in charge of the affected forest for sacrifice to appease the gods and prevent calamitous consequences. Such serial calamities commence with the offender, moves to members of his immediate family, the extended family and then other community members and stops only when the required sacrifices have been done.

A taboo in Nigeria prevents the use of wooden baton to remove the bark of certain important medicinal plants. Under no circumstance should a metal be used on some other plants. A particular forest may be closed for exploitation of medicinal plants and other resources at certain times of the year when some very important festivals are on. Such festivals can last up to four months thus giving enough time for regeneration of some exploited plants. As different forests are closed at different times of the year, procurement of medicinal plants is not totally stopped. The preservation of many important trees such as *Garcina kola*, *Enantia chlorantha, Okoubaka aubrevielli, Melicia excelsa, Bombax buonopozense *and *Ceiba pentandra *in forests or on farmlands have been made possible through the belief that these plants harbour some spirits of a community' ancestors and as such should not be cut down without first appeasing these spirits. Such trees have white or red cloth tied round the base and are usually points for conducting rituals, ceremonies and sacrifice presentation. The materials needed for appeasing the spirits are usually very difficult to procure (e.g. an animal of specified age with totally white or totally black fur without spots or wrinkles) and this presents a barrier to indiscriminate felling of such trees [2,12,38,39].

Rather interestingly, many of the remedies used in TM have been evaluated by scientists and evidence from research confirms the efficacy claimed for certain herbal remedies [10,17,40]. Such research requires the setting-up of an animal model where possible, and testing the herb or component thereof in the particular form in which they are used or in close approximation (alcohol extract where palm wine or local gin is used). The basic objective of such research is establishing that some of the practices and remedies used in TM are not totally useless or worse still, without scientific bases. For example, it has been found that most of the traditional medicaments used to heal wounds possess some anti-microbial and anti-inflammatory properties [10]. Some of these TMs are now being packaged in more acceptable forms, subjected to laboratory tests and given approval for public use by the National Agency for Food and Drug Administration and Control in Nigeria [41].

## Economic considerations in the use of TM

Average household income in most of Sub-Saharan Africa (SSA) is extremely low compared to that of other developing and developed economies. For example, the per capita income for Nigeria is estimated at $560 as against $37740, $43560, $38950, $15840 and $4770 for UK, USA, Japan, Korea and South Africa, respectively. There is a significant level of poverty in SSA and, especially, Nigeria. Per capita consumption expenditure is less than USD1.00 per day [42]. The level of poverty has excluded a significant proportion of the population, especially in the rural locales, from accessing modern health care facilities even when it is available. Poor access to OM is also compounded by very low public expenditure on health, amounting to about USD14.00 per person per year. The inadequacy of public health infrastructure is one of the major reasons for poor quality of service in public health institutions. Consequently, the infant mortality for the region is reported to be 55% higher and life expectancy about 11 years lower than the rest of the world's low-income developing countries. Maternal mortality is double that of other low- and middle-income developing countries [43]. Private healthcare facilities are comparatively more efficient in terms of quality of service but charge fees that are far beyond the level the average farming household could afford. Many rural and farming households would therefore be without any form of healthcare facility without the TM [12]. The combination of high level of poverty, inadequate public health infrastructure and high cost of private health care services has confined the larger proportion of both the urban and especially the rural populace to patronizing TM [5,11,12].

The most common ailment affecting the people is malaria which has been reported as leading to drastic reduction in agricultural productivity [9,44,45]. According to the WHO [46], malaria is now resistant to Chloroquine therapy. The recommended Atesunate Combination Therapy (ACT) is hardly ever offered free in most public healthcare facilities. The average cost of malaria treatment based on ACT is estimated to be about N1,500 (USD 10.00) inclusive of cost of laboratory tests. This is a princely sum for the average Nigerian in the rural areas which are characterized with low household incomes [8,47-49]. A TM therapy for the same ailment will cost on the average N200 or could even be procured for free, if the person could collect the medicinal plants and prepare the medicament personally. When the ailments are complicated such as internal organs mal-functioning, mental illness or barrenness, the cost implications are far beyond the income capacity of the average citizen. In such instances, TM is the only option available for obtaining some remedy at affordable costs. Hence, a large proportion of Nigerians, especially those living in rural areas, continues to patronize TM [5]. TM continues to grow because traditional healers are considered successful in curing a large number of illnesses [2,23].

In summary, the reasons adduced for the rise in the uptake of TM in Nigeria are:

1. Inadequate modern medical practitioners. The ratio of TM practitioners to the total population is estimated at 1:250 while that of OM practitioners is 1:26,000 [50-52].

2. High and rising proportion of fake and adulterated synthetic drugs which makes a lot of people to crave for natural products [53].

3. The resistance of some pathogenic organisms to synthetic drugs (e.g. *Plasmodium falciparum *to chloroquine).

4. Facilities are inaccessible for much of the population. In some urban areas the average waiting time at a hospital or clinic can be as much as eight hours.

5. The staff are poorly trained and ill-motivated.

6. Many staff members, believing they hold superior knowledge, treat patients inconsiderately.

7. Patients are frequently not told the nature and causes of their illnesses.

8. There are inadequate technical services leading to poor quality care.

9. The treatment costs too much, even in public hospitals and clinics.

10. Governments spend a large proportion of the per capita GNP on OM.

11. Treatment is divorced from the patient's culture, family and community.

12. The treatment only addresses a patient's biological manifestation of the illness and does not attempt to heal spiritual aspects of illness [52].

Arising from increased use, the practice of TM is now being modernized and popularized in Nigeria culminating in increasing number of herbal homes and growing confidence in TM. A crop of educated practitioners now exploits plants for large-scale production of TM products. The hitherto unregulated activity is now being checked to maintain standard by government agencies to eliminate quacks. There is increasing number of NAFDAC-certified TM products being sold to the public [41]. To complement this effort, State Governments have also come up with Traditional Medicine Councils to oversee the affairs of practitioners. These steps are serving to instill the confidence in many Nigerians to patronize TM. If the present trend in patronage continues, Nigeria may soon be ranking favourably with China, India and Korea in terms of the proportion of her GDP accruing from TM. The constraints to achieving this are the low level of herbal knowledge transfers to the succeeding generation of Nigerians and rapid bio-diversity loss arising from scientifically unsustainable methods of harvesting medicinal plants [2,17].

## Conclusion

Despite the deficiencies inherent in TM and the funding of OM to the exclusion of TM by governments in Nigeria, a number of factors are at present compelling an increasing number of people to turn to TM for solutions to their health problems. The fact that TM practitioners in Nigeria are often uneducated and had it handed down to them by informal and sometimes supernatural means has made the practice to be closely interwoven with their culture and beliefs. This probably explains the emergence and observance of some socio-cultural and supernatural or magico-religious practices which have become part and parcel of the practice of TM in Nigeria. Some of these practices can be explained with the principles of modern science and are therefore in conformity with the practices in OM. Others go beyond the realms of ethno-botanical research as their roles in the potency of medicines prepared for use cannot be proved. It is believed that there are medicinal preparations that will not be potent without the observance of these practices even if all active ingredients are present. A popular example is the role of incantations in the treatment of complicated cases of ill-health. Some OM practitioners and other educated elites however dismiss some of these practices as mere manipulations of the psychology of the patients by the TM practitioners.

There is a need for increased oversight function on TM in Nigeria which, among others, should streamline practices and minimize variations which are tied to the local culture and beliefs of the practitioners. This is a *sine-qua-non *for the integration and co-recognition of the TM and OM systems of healthcare. This step will necessitate the training of TM practitioners to improve their skills and make them eliminate practices which they truly know have no implications for the potency of their medicaments. This will further enhance their utilization of the referral system in dealing with high-risk cases. There should also be the willingness on the part of health policy makers in Nigeria to gain a better understanding of TM and fund research activities to evaluate them. This will help identify various practices that can be adapted into OM system and those that are unnecessary or harmful that should be discouraged in line with the suggestion by Odugbemi [54].

## Declaration of Competing interests

The authors declare that they have no competing interests.

## Authors' contributions

TE conceived the study, collected and read up the literature materials needed and coordinated the drafting of the manuscript. AE took part in reading some of the literature materials and manuscript drafting and revision. All authors read and approved manuscript.

## Authors' informations

TE holds a PhD in Agricultural Economics and is a Senior Lecturer in the Department of Agricultural Economics & Extension, The Federal University of Technology, Akure, Nigeria. He is also Acting Associate Director (Consulting) in the University's Centre for Research & Development, a position which widens his opportunity of working with peasant farmers on poverty-reduction projects all over the country. His experience of working with poor farmers, who are constantly in search of good health and cost-reducing production techniques, led to the idea for this paper. TE is the son of a traditional ruler in Nigeria who by tradition is the custodian of the cultural beliefs and practices of his people. He grew up in the palace and observed some of the TM practices first-hand.

AE holds a PhD in Agricultural Economics and is an Associate Professor in the same department and university as TE. He was a one-time Acting Director of the Centre for Research & Development of The Federal University of Technology, Akure, Nigeria. He is widely experienced in handling rural development projects directed at enhancing the welfare of small farmers.
